# Supplementary material for: Pyruvate Dehydrogenase Kinase 1 inhibition mediated oxidative phosphorylation enhancement in cartilage promotes osteoarthritis progression
Source: BMC Musculoskelet Disord. 2023 Jul 20;24:597. doi: 10.1186/s12891-023-06585-6 (PMC10357736; doi:10.1186/s12891-023-06585-6)
Supplement: Supplementary file 1 — Additional file 1. [file 12891_2023_6585_MOESM1_ESM.zip › MF.Gsea.1653627316242/GOMF_CULLIN_FAMILY_PROTEIN_BINDING.html]

Details for gene set GOMF\_CULLIN\_FAMILY\_PROTEIN\_BINDING[GSEA]

|  || Dataset | 55457\_collapsed\_to\_symbols.cls.cls #OA\_versus\_normal.cls.cls #OA\_versus\_normal\_repos |
| Phenotype | cls.cls#OA\_versus\_normal\_repos |
| Upregulated in class | OA |
| GeneSet | GOMF\_CULLIN\_FAMILY\_PROTEIN\_BINDING |
| Enrichment Score (ES) | 0.37530297 |
| Normalized Enrichment Score (NES) | 1.1715693 |
| Nominal p-value | 0.2264574 |
| FDR q-value | 0.60835576 |
| FWER p-Value | 1.0 |
Table: GSEA Results Summary

  

Fig 1: Enrichment plot: GOMF\_CULLIN\_FAMILY\_PROTEIN\_BINDING      
 Profile of the Running ES Score & Positions of GeneSet Members on the Rank Ordered List

  

| SYMBOL | TITLE | RANK IN GENE LIST | RANK METRIC SCORE | RUNNING ES | CORE ENRICHMENT || 1 | RBX1 | ring-box 1 [Source:HGNC Symbol;Acc:HGNC:9928] | 348 | 0.724 | 0.1119 | Yes |
| 2 | KLHL36 | kelch like family member 36 [Source:HGNC Symbol;Acc:HGNC:17844] | 796 | 0.570 | 0.1867 | Yes |
| 3 | KLHL9 | kelch like family member 9 [Source:HGNC Symbol;Acc:HGNC:18732] | 1006 | 0.516 | 0.2694 | Yes |
| 4 | DCUN1D1 | defective in cullin neddylation 1 domain containing 1 [Source:HGNC Symbol;Acc:HGNC:18184] | 1798 | 0.385 | 0.2818 | Yes |
| 5 | PRKN | parkin RBR E3 ubiquitin protein ligase [Source:HGNC Symbol;Acc:HGNC:8607] | 1972 | 0.364 | 0.3382 | Yes |
| 6 | DDB1 | damage specific DNA binding protein 1 [Source:HGNC Symbol;Acc:HGNC:2717] | 2302 | 0.326 | 0.3753 | Yes |
| 7 | RNF7 | ring finger protein 7 [Source:HGNC Symbol;Acc:HGNC:10070] | 3264 | 0.225 | 0.3438 | No |
| 8 | KCTD5 | potassium channel tetramerization domain containing 5 [Source:HGNC Symbol;Acc:HGNC:21423] | 4638 | 0.101 | 0.2566 | No |
| 9 | BTBD1 | BTB domain containing 1 [Source:HGNC Symbol;Acc:HGNC:1120] | 4799 | 0.087 | 0.2610 | No |
| 10 | KCTD17 | potassium channel tetramerization domain containing 17 [Source:HGNC Symbol;Acc:HGNC:25705] | 5239 | 0.053 | 0.2371 | No |
| 11 | CCDC22 | coiled-coil domain containing 22 [Source:HGNC Symbol;Acc:HGNC:28909] | 5493 | 0.033 | 0.2238 | No |
| 12 | KCTD9 | potassium channel tetramerization domain containing 9 [Source:HGNC Symbol;Acc:HGNC:22401] | 6901 | -0.072 | 0.1284 | No |
| 13 | KCTD2 | potassium channel tetramerization domain containing 2 [Source:HGNC Symbol;Acc:HGNC:21294] | 7793 | -0.141 | 0.0863 | No |
| 14 | SKP1 | S-phase kinase associated protein 1 [Source:HGNC Symbol;Acc:HGNC:10899] | 8454 | -0.190 | 0.0716 | No |
| 15 | KLHL21 | kelch like family member 21 [Source:HGNC Symbol;Acc:HGNC:29041] | 9668 | -0.289 | 0.0328 | No |
| 16 | DCUN1D4 | defective in cullin neddylation 1 domain containing 4 [Source:HGNC Symbol;Acc:HGNC:28998] | 10518 | -0.375 | 0.0390 | No |
| 17 | DCUN1D2 | defective in cullin neddylation 1 domain containing 2 [Source:HGNC Symbol;Acc:HGNC:20328] | 10538 | -0.377 | 0.1098 | No |
| 18 | KLHL3 | kelch like family member 3 [Source:HGNC Symbol;Acc:HGNC:6354] | 10592 | -0.383 | 0.1792 | No |
Table: GSEA details [plain text format]

  

Fig 2: GOMF\_CULLIN\_FAMILY\_PROTEIN\_BINDING      
 Blue-Pink O' Gram in the Space of the Analyzed GeneSet

  

Fig 3: GOMF\_CULLIN\_FAMILY\_PROTEIN\_BINDING: Random ES distribution      
 Gene set null distribution of ES for **GOMF\_CULLIN\_FAMILY\_PROTEIN\_BINDING**
